# Balance Dysfunction in Parkinson's Disease

**DOI:** 10.1155/2015/434683

**Published:** 2015-01-14

**Authors:** Steno Rinalduzzi, Carlo Trompetto, Lucio Marinelli, Alessia Alibardi, Paolo Missori, Francesco Fattapposta, Francesco Pierelli, Antonio Currà

**Affiliations:** ^1^Neurology and Neurophysiopathology, Sandro Pertini Hospital, Via Monti Tiburtini 385, 00157 Rome, Italy; ^2^Institute of Neurology, Department of Neurosciences, Rehabilitation, Ophthalmology, Genetics, Maternal and Child Health (DiNOGMI), Largo Daneo 3, University of Genova, 16132 Genova, Italy; ^3^Academic Neurology Unit, A. Fiorini Hospital and Department of Medical-Surgical Sciences and Biotechnologies, Sapienza University of Rome, Polo Pontino, Via Firenze, 04019 Terracina, Italy; ^4^Neurosurgery Unit, Policlinico Umberto I, Department of Neurology and Psychiatry, Sapienza University of Rome, Via del Policlinico, 00161 Roma, Italy; ^5^Neurology Unit, Policlinico Umberto I, Department of Neurology and Otolaryngology, Sapienza University of Rome, Via dell'Università 30, 00185 Roma, Italy; ^6^Academic Neurorehabilitation Unit, ICOT and Department of Medical-Surgical Sciences and Biotechnologies, Sapienza University of Rome, Polo Pontino, Via F. Faggiana 34, 04100 Latina, Italy; ^7^INM Neuromed IRCCS, Via Atinense 18, 86077 Pozzilli, Italy

## Abstract

Stability and mobility in functional motor activities depend on a precise regulation of phasic and tonic muscular activity that is carried out automatically, without conscious awareness. The sensorimotor control of posture involves a complex integration of multisensory inputs that results in a final motor adjustment process. All or some of the components of this system may be dysfunctional in Parkinsonian patients, rendering postural instability one of the most disabling features of Parkinson's disease (PD). Balance control is critical for moving safely in and adapting to the environment. PD induces a multilevel impairment of this function, therefore worsening the patients' physical and psychosocial disability. In this review, we describe the complex ways in which PD impairs posture and balance, collecting and reviewing the available experimental evidence.

## 1. Introduction

Parkinson's disease (PD) is a common neurodegenerative disorder, affecting middle aged and elderly people. It is a disease characterised by dopaminergic and nondopaminergic deficiency [1, 2] causing a variety of nonmotor symptoms such as sensory symptoms (pain and tingling), hyposmia, sleep alterations, depression and anxiety, abnormal executive and working memory-related functions, and motor symptoms such as bradykinesia, rigidity, tremor, and disturbance of postural control.

Among the motor symptoms, those arising from disturbance of postural control—either static or dynamic—are complex and not entirely understood. Subtle postural changes become evident shortly after the onset of the illness. The most recognised type of static deformity is the classic stooped simian appearance, with flexion of the hip and knees and rounding of the shoulders. More severe abnormalities of static posture disrupting spinal alignment and leading to significant disability include camptocormia, antecollis, Pisa syndrome, and scoliosis [3, 4].

In the late stages of the disease, when the postural reactions begin to be impaired [5] or inadequate [6], patients manifest abnormal dynamic postural control (i.e., postural instability). This is a highly disabling symptom because it is poorly controlled by dopaminergic therapy; if present early in the disease, a form of atypical Parkinsonism should be suspected [7].

The clinical examination of static posture is made by inspection, whereas control of dynamic posture is limited to the evidence of thrust (pull test), in which the physician stands behind the patient and briskly pulls him backwards by the shoulders. This test is considered positive if the patient takes more than two steps to regain balance, or if he falls if unsupported by the examiner. Clinically, postural instability therefore translates in loss of balance control.

The term “balance control” refers to a multisystem function that strives to keep the body upright while sitting or standing and while changing posture. Balance control is needed to keep the body appropriately oriented while performing voluntary activity, during external perturbation, and when the support surface or environment changes. Faulty balance control mechanisms may contribute to fall-related injuries, restriction of gait patterns, and decreased mobility. These disabilities lead to loss of functional independence and social isolation.

Balance control is assured through dynamic control of posture, which in turn is exerted by generating postural responses to perturbations. Normally, such responses are generated by automatic mechanisms that contribute to the maintenance of upright posture and prevent the subject from falling. Postural perturbations determine the activation of the sensory systems, integration at the level of the central nervous system, and formulation of a motor response aimed at maintaining the body's centre of gravity within the base support of the subject [8]. Theoretically, in patients with Parkinson's disease, postural instability may be the result of faulty processing in three main distinct processes:

- sensory organization, in which one or more of the orientational senses (visual, vestibular, and somatosensory) are involved and integrated within the basal ganglia,
- motor adjustment process, which provides a properly scaled neuromuscular response,
- background muscle tone, known to be hypertonic in Parkinsonian patients.

## 2. Sensory Organization: Visual, Vestibular, and Somatosensory Inputs

Postural control in humans depends on the information coming from visual, vestibular, and somatosensory systems. As it happens for voluntary movement, during postural movements the somatosensory input is generated by muscle sensory organs, mainly spindles (sensitive to changes in fascicle length), and Golgi tendon organs (sensitive to changes in muscle tension) [9–12]. Proprioceptive information is integrated with visual and vestibular information in order to interpret the complex sensory environment, and to weigh the relative dependence of posture on each of the senses. When tested while standing on a firm support base, in a well-lit environment, a healthy person's balance control relies on somatosensory, visual, and vestibular information [13]. In this condition, vestibular receptors detect accelerations and deviations in head orientation, visual sensors detect eye-head orientation related to the visual world, and proprioceptors mainly detect foot flexion in relation to the support surface. During upright stance, vestibular, visual, and proprioceptive cues are combined, because each sensory system detects the body sway from a reference position, for the various body segments. The central nervous system sums up the individual sensory signals and, as a function of these combined signals, generates an appropriate corrective motor response [13, 14].

Patients with PD may develop a range of visual problems during the course of the disease. Changes in vision may result from alterations in visual acuity, contrast sensitivity, colour discrimination, pupil reactivity, eye movements, motion perception, visual field sensitivity, and visual processing speed. Slower visual processing speed can also lead to a decline in visual perception especially for rapidly changing visual stimuli. In addition, there may be disturbances of visuospatial orientation, facial recognition problems, and chronic visual hallucinations [15]. Visual deficits in PD are considered important in influencing overall motor function [16, 17]. As in normal subjects, in patients with PD, visual control is needed to stabilize posture by reducing the spontaneous oscillations and changing the postural strategy implemented to maintain the centre of gravity of the body within the support base [18]. Studying postural responses after visual stimuli generated by discrete lateral displacements of a moveable room in which subjects stand in the upright position, PD subjects showed normal sway with eyes opened or closed but produced disproportionately large motor responses to room movement, which did not attenuate with stimulus repetition [19]. This observation suggests that PD subjects are overreliant on visual information, and this was interpreted by the authors as indicating overactivation of a visual-postural circuit involving the basal ganglia, concerned with the reweighting of the various sensorimotor loops controlling posture in the process of adapting to novel situations [19].

Overreliance of posture control on the visual channel might represent a compensatory mechanism to the deficit of other sensory systems rather than a primary alteration [20]. Studying arm movements alone or in combination with a forward bending of the trunk, with or without visual feedback, shows that OFF-therapy PD subjects make as accurate reaches as controls with or without vision, but unlike controls, they are unable to synchronize the fingertip and trunk motions [21]. Visual feedback reduced the variability in fingertip-trunk timing, an effect not observed in healthy subjects, whose timing proved to be independent of vision. This finding extends previous kinematic observations showing how visual feedback improves the motor performance in PD patients in terms of movement initiation, movement trajectories, and accuracy [22, 23].

Tilting reactions are abnormal in PD, suggesting that the labyrinthine postural reactions undergo central integration in the basal ganglia [3]. Activation of the vestibular system can be made also by galvanic vestibular stimulation (GVS), which allows modulation of vestibular nerve firing rate. When applied to an upright subject, bimastoid GVS induces postural sway, according to head position, towards the side of the anodal electrode [24]. PD subjects as a group produce postural responses to GVS of normal amplitude, latency, and direction of the induced body sway, but when they were subdivided into two subgroups according to a clinical assessment of postural deficit, the more disabled subgroup responded with increased body speed than either controls or the mildly affected patient subgroup. The authors concluded that postural deficits in mildly or moderately affected PD patients are not explained by vestibular dysfunction [25]. Using bicathodal stochastic GVS to influence the body sway during upright stance with eyes opened or closed, Parkinsonian subjects showed small reductions in sway in the “eyes closed” condition at low current intensity of stimulation [26]. As stochastic GVS induces stochastic resonance—in which a noise of randomly changing intensity enhances detection of subthreshold stimuli—the proposed mechanism for stochastic GVS benefit in PD subjects was the enhanced detection of small changes in posture signaled by vestibular afferents, allowing for early compensatory postural adjustments and thus reducing body sway [26].

Studies of the somatosensory system in the control of posture in PD subjects mainly used stimuli capable of activating the muscle spindles and evaluating kinesthetic function. Kinaesthesia is defined as the conscious perception of active or passive motion and direction of movements. It relies on the processing of proprioceptive information derived mainly from muscle spindles and joint and cutaneous receptors. During everyday life activities the subject has no awareness of kinaesthesis but is still able at any time to locate the position of his body in relation to space, even in the absence of visual information [11, 27].

When PD subjects are asked to point to a target in the upright position with their eyes opened or closed, they show normal pointing accuracy but reduced body centre of mass displacements when moving with eyes closed [28]. The selective effect of eye closure on the centre of mass displacement but not on pointing accuracy means that the pointing and postural components of the task relate to separate motor programs. Decoupling of task components in Parkinsonian subjects but not in controls suggests that the basal ganglia are involved in the integration of proprioceptive information for posture-movement coordination. However, this should be considered as an oversimplification, because findings from a study evaluating the relation of motor asymmetries to perceptual asymmetries during a perceptuomotor task in Parkinsonian subjects [29] showed that patients had a left bias in both baseline pointing and pointing during trunk rotation. Such perceptual lateralization of the peripersonal space is independent of the side of disease onset, disease progression, or the medication status, therefore suggesting variable asymmetry in the wider central representation of the bilateral neural processes that balance afferent sensory input from both sides of space and body to form representations referenced to the body midline when generating movements to interact with perceived external hemispace.

In subjects with PD, several studies have demonstrated altered kinaesthesis of the upper limbs, head, and of the trunk. In the upper limbs and head the lowest range of movement perceivable by the Parkinsonian subjects is higher than that of controls, and the ability to identify the direction of movement is reduced [27, 30]. As a consequence, to be correctly perceived by the subjects, the movement of a limb must have a larger joint excursion than in controls. Kinesthetic changes correlate with the most affected side in the case of asymmetric symptoms, with the disability score according to the* Unified Parkinson's Disease Rating Scale* (UPDRS), and with the duration of disease. The fact that kinesthetic abnormalities are found in PD but not spinocerebellar subjects [27] suggests that an intact corticobasal ganglia loop is essential for awareness of the limb position and that the basal ganglia play a selective role in kinaesthesis. Abnormal kinaesthesis of axial musculature in subjects with PD has been shown by studying the perception threshold and the ability to discriminate the direction of the passive movement of the pelvis compared to the foot (hip kinaesthesis) and of the hip compared to the shoulder (trunk kinaesthesis) [31]. Interestingly, levodopa proved to have a negative impact on axial kinesthesia, insofar as abnormalities observed in OFF medication phase worsened when ON.

Since the basal ganglia are involved in the perception of motion and its direction and proprioception plays an important role in the control of postural reactions evoked by unexpected stimuli, it is reasonable that in PD kinesthetic defects may impair the patients' ability to perform postural responses “adequate" to external perturbations, therefore contributing to postural instability.

## 3. Postural Reactions and the Scaling of Postural Response

In humans, the sudden perturbation to a supporting surface induces loss of stability in standing posture. In order to regain perturbed balance, the muscles of the lower limbs contract automatically. The onset of activation of muscle contraction is shorter than voluntary reaction times but longer than the monosynaptic loop time. Therefore, postural reactions are generally considered automatic, which falls somewhere within the spectrum between reflexive and volitional. Posture is not purely reflexive because even in the earliest postural reactions—likely activated by somatosensory information—it is not always the stretched muscles that are activated. In addition, postural reactions involve activation of muscle synergies throughout the entire body; they depend on prior experience and may change according to task and context, thereby proving more flexible and adaptable than spinal proprioceptive reflexes.

Neurophysiological study of the muscular-automatic activity evoked by the movement of the support surface has revealed a series of responses recorded from the lower limbs. Sudden toe-up tilts in pitch or forward sway perturbations of a supporting platform produce a distal-to-proximal muscle EMG activation pattern consisting of a short (SLR 30–60 ms)- and a medium-latency response (MLR 70–120 ms) recorded from medial gastrocnemius muscle, as well as a long-latency response (LLR 100–200 ms) recorded from tibialis anterior and vastus medialis muscle. The short- and medium-latency responses generated in the stretched muscles destabilize the subject further, whereas the long-latency response in the shortened antagonist muscles contributes to postural stabilization [32, 33]. This activation pattern exerts compensatory torques about the ankle joints, which restore balance control by moving the body centre of mass forward or backward [34].

Reasonably, abnormal generation of motor patterns, such as delayed onset of muscle activation, inappropriate amplitude, and reversal of the normal activation sequence produce less effective correction of destabilized posture. Indeed, when subjected to postural perturbations, PD subjects show increased amplitude of the MLR, with response amplitude correlating to disease severity, and anticipated LLR onset latency [5, 35]. Parkinsonian subjects studied under static (quiet stance) and dynamic (i.e., displaced by movement of a supporting platform) conditions during free and supported stance [35] manifested peculiar features at posturographic analysis. Under static conditions, they simply shifted their center of foot pressure, the abnormal position of which moves from backwards to forwards according to disease severity. Under dynamic conditions, postural responses to perturbations of free stance showed increased magnitude of MLRs and LLRs that proved unrelated to the disease stage; the position of the center of foot pressure changed differently and selectively with the magnitude of early and late muscle responses according to direction of translation (i.e., forward) and of toe-rotation (i.e., up). Under supported conditions Parkinsonian subjects progressively failed to suppress MLRs and LLRs to perturbation (as it happened in controls) with increasing disease severity. This pattern of EMG activation means that the normal distal to proximal muscle activation sequence is reversed and that contraction of the hip muscles precedes that of ankle muscles thus increasing the limb stiffness and inducing lack of appropriate corrective movements. Finally, because MLRs further destabilize posture, their amplitude increasing with disease severity establishes an association between automatic postural responses and clinically rated balance control in PD and also suggests that the basal ganglia exert a modulatory influence on the MLR circuit [5, 36].

Automatic postural responses to support platform displacement have also been investigated by studying the surface reactive torque and EMG activity in leg and trunk muscles. Typical EMG response pattern to backward or forward surface translations in a representative standing young subject is a burst of EMG activation in “dorsal” (i.e., gastrocnemius, hamstrings, paraspinal, GAS-HAM-PSP) and “ventral” muscles (i.e., tibialis anterior, quadriceps, hip abductors, TIB-QUAD-ABD) in a distal-to-proximal pattern with silent antagonists. This proximal-to-distal activation always resulted in reciprocal activation at the trunk muscles. In contrast, Parkinsonian subjects coactivate proximal muscles on the ventral side of the body (QUAD-ABD) with backward surface translation, and on dorsal side of the body (PSP and HAM) with forward surface translation. The excessive antagonist muscle activation in Parkinsonian subjects was associated with reduced surface-reactive torque, less passive body sway, and abnormally coordinated inflexible postural response [6].

To maintain equilibrium, postural responses to surface perturbations must be appropriately scaled to both how fast and how far the center of body mass is displaced on the base of support. A basic abnormality of voluntary upper limb movements in PD is the patients' inability to scale the size of the first agonist burst for the required movement displacement [37]. Similarly, Parkinsonian patients are unable to scale the size of their LLR in response to postural perturbations [38].

Posture may be perturbed by a variety of stimulation procedures, and scaling the postural response needs to take into account the characteristic of the stimulus and how they change to challenge posture. One perturbation procedure is to transiently vibrate leg muscles in standing subjects. Muscle vibration produces selective activation of muscle proprioceptors and low-threshold afferents (Ia fibers) and thus induces postural responses and postural kinesthetic illusions. As PD patients with postural instability are known to hyperreact to visual, vestibular, and proprioceptive sensory manipulation of the neck muscles [39], it was thought that a similar abnormality in leg muscles might alter the scaling of postural response. Therefore, early-stage and advanced-stage PD subjects were subjected to static posturography with eyes closed, while their soleus muscles received short bursts of mechanical vibration. Normally, leg muscle vibration during stance induces an early forward response beginning at 90 ms and peaking around 300 ms and a late response beginning at 500 ms and peaking around 2000 ms. Compared to controls, advanced-stage PD subjects showed responses of normal latency but with increased amplitude. The fact that severely affected PD subjects swayed significantly more during artificial modification of the proprioceptive signal than did mildly affected subjects and healthy controls suggests that impairment in scaling of postural response is a generalized defect, which involves also the afferent side of the process, that is, scaling sensory information [40].

More recently, a feedback control model of body dynamics has been developed to investigate whether the postural impairments of PD could be described as an abnormal scaling of postural feedback gain [41]. The model assumes that the nervous system takes body dynamics into account and adjusts postural feedback gains to accommodate biomechanical constraints. Body dynamics are inferred from ground reaction force and kinematic data recorded by markers located at the neck, shoulder, hip, knee, ankle, toe, heel, and the platform surface. Feedback gains quantify how the nervous system generates compensatory joint torques by actively controlling joint stiffness and damping, when subjects experience surface translation based on kinematic responses. When PD subjects are subjected to backward translations, the model shows that they have smaller than normal ankle feedback gain with low scaling and larger hip feedback gain. This coupling leads to “bradykinetic” postural responses and early violation of the flat-foot constraint, that is, the biomechanical constraint that determines the maximum allowable ankle torque for upright stance while keeping the feet flat on the floor. Indeed, in PD patients responding to large perturbations, the low ankle gain together with the inappropriately low scaling induces greater ankle joint torques than what is biomechanically allowable, a phenomenon that results in early onset of heel lift or stepping. Reasonably, this is the mechanism explaining why PD patients use premature compensatory stepping responses to shoulder pull [41].

To see whether and which abnormality of automatic postural responses depends on acute dopamine deficiency, the leg muscle EMG responses induced during stance by impulsive forward or backward displacements recorded in a group of PD subjects were compared to those of age-matched healthy subjects studied before and after intake of a dopamine antagonist (haloperidol) [42]. Both Parkinsonian subjects and haloperidol-treated subjects showed smaller than normal compensatory gastrocnemius EMG responses to backward displacements. In both groups, the inability to compensate for the perturbations correlated to reduced sensitivity of the gastrocnemius muscle to stretch, but only in PD subjects the gastrocnemius response was followed by enhanced activation of the tibialis anterior muscle (thus indicating this was not due to acute dopamine deficiency). In addition, Parkinsonian subjects showed slower than normal angular rotation at the ankle joint induced by faster backward-direct displacement, despite similar gastrocnemius EMG activity. These findings suggest changes in the inherent muscle stiffness in PD [42].

Dopaminergic medication fails to improve balance control in PD possibly because it corrects early and late automatic postural responses only partially and distinctly [43]. In this study, standing Parkinsonian subjects received 4 degrees “toe-up" rotational perturbations of a supporting force plate, while their MLR and LLR were recorded from antagonist leg muscles, and the position of their center of gravity was assessed. During the OFF phase, PD subjects showed increased MLR and reduced LLR, together with a markedly increased posterior displacement of the center of gravity. Clinically these data indicate that the initial forward destabilizing displacement is increased, while the subsequent backward displacement (a measure of the functionally corrective braking action of LLR) is delayed. During the ON phase LLR amplitudes increased modestly; conversely, MLR amplitudes decreased, even though remaining comparatively higher than in controls, with no benefit on the displacement of the center of gravity. Therefore, dopaminergic medication improved the destabilizing displacement only partially while it left later postural corrections substantially unchanged. From that, it is arguable that the abnormal posterior displacement of the center of gravity induced by rotational perturbations of a supporting force plate in Parkinsonian subjects does not benefit from dopaminergic medication.

The effect of dopamine replacement therapy on the ability of scaling the magnitude of automatic postural responses based on somatosensory feedback or on predictive central set was also investigated [44]. Acute dopaminergic replacement induced various changes, lowered further the magnitude of initial torque and EMG responses, worsened scaling based on the central set, and left the scaling based on sensory feedback unchanged. Finally, dopaminergic medication did not revert increased sway in response to muscle vibration in advanced-stage PD subjects.

## 4. Influence of Peripheral and Central Drive on Generating Automatic Postural Responses

Automatic postural responses depend on the influence that peripheral and central drive exert on the generated motor response. “Central drive” refers to the descending commands that prepare sensory and motor systems for anticipated stimulus and task conditions [45]. The advantage to set the response in advance is to speed up and optimize the motor response to the significant stimulus; the disadvantage is that the central set produces errors in the motor responses when the stimulus or external conditions change unexpectedly [46].

The contribution of the central drive set to peripherally triggered motor responses is revealed by analyzing responses generated when the characteristics of the stimulus perturbation remain unchanged but the subject's expectations of stimulus characteristics vary. For example, postural responses to a certain perturbation differ if identical perturbation characteristics are presented repeatedly (expected condition) rather than randomly (unexpected condition), or in combination with other types of perturbations. When the characteristic of a repeated perturbation varies unexpectedly, errors appear in the early components of the motor response, and these changes have been attributed to central set effects [47, 48].

In normal subjects exposed to backward translations of the support surface causing forward sway, not only are torque and EMG response magnitudes scaled by peripheral sensory information that code the velocity and amplitude of the displacement [49], but they are also modulated by central set on the basis of prior experience [50]. In this study, the scaling of the initial agonist responses disappeared when perturbation amplitudes were randomized, and subjects underresponded when they expected small perturbation. The central set influenced the magnitude but not the onset of early muscle responses to perturbation, whereas late responses better showed up after perturbation of unexpected characteristics.

The fact that descending motor commands influence the generation of postural responses to perturbation in Parkinsonian subjects was also suggested by the observation that, after perturbation of free and supported stance, the position of the center of foot pressure changed differently and selectively with the magnitude of early and late muscle responses [35]. Indeed, the authors interpreted the amplitude modulation of muscle responses for perturbations of free stance as a compensatory adaptation to the abnormal upright posture and the inappropriate suppression of the late responses during supported stance as indicating failure of motor program selection.

Detailed experiments were designed to investigate whether PD affects the scaling of automatic postural responses differentially when these are generated based on somatosensory feedback or on a predictive central set. Automatic postural muscle responses were recorded from posturally unstable Parkinsonian subjects standing on a supporting platform and subjected to sudden toe-up rotations [38]. Central set was manipulated by varying the perturbation amplitude either predictably or unpredictably. This investigation revealed that not only Parkinsonian subjects failed to scale the size of their LLR to predictable changes in perturbation amplitude, but they also lacked the ability to modulate LLR during unpredictable serial perturbations. Since patients proved to be disabled in both scaling of automatic response and shifting of central set, it was concluded that the patients' inability to modify LLRs may be a factor contributing to postural instability in PD [38].

A later study aimed to evaluate the effects of parkinsonism and of dopamine replacement therapy on the scaling of automatic postural responses based on peripheral sensory information and on central set, compared measures of surface reactive torques and EMG activity in response to backward surface translations in Parkinsonian patients and elderly controls [44]. The scaling of the magnitude of postural responses generated using somatosensory feedback was quantified by analyzing the correlation between the initial rate of change of torque, the integrated EMG, and the translation velocity during unexpected perturbations. The same correlations obtained during expected perturbations were used to quantify the scaling of postural responses generated on the basis of predictive central set. The findings confirmed that automatic postural responses in Parkinsonian subjects were never delayed, but rather anticipated (in antagonist quadriceps), which led to muscle coactivation at the knee. Parkinsonian postural responses were characterized by fragmented EMG activity with multiple bursts of short duration, small agonist extensor bursts, and large antagonist flexors bursts. Patients scaled postural responses to both displacement velocities and amplitudes, but their torque responses were smaller than those of controls, especially for the largest perturbations displacement. Although Parkinsonian subjects had smaller torques, the slope of their postural response magnitude scaling to displacement velocity was normal. When perturbation displacement was predictable, Parkinsonian subjects proved able to scale postural responses only for small expected displacement, whereas they were unable to scale plantar flexion torques to perturbation of increasing displacement amplitudes. In these conditions, they generated reduced torque associated with reduced agonist- and increased antagonist-muscle activity, leading to cocontraction. Overall, during backward translations Parkinsonian patients scale EMG activity to produce motor responses to postural perturbation, but their scaled muscular activation is fragmented and produces less torque than normal. Such abnormality predominates for perturbations of increasing displacement and takes place for all postural responses, that is, generated using peripheral information or a central set, suggesting that the main postural deficit of PD patients is their inability to quickly generate an adequate level of postural force [44].

Interesting clues to clarify the nature of balance control deficits in PD come from studies investigating patients affected by distinct neurodegenerative diseases [51, 52]. These studies compared patients affected by Alzheimer's disease (AD) or PD in their ability to maintain upright balance under varying sensory conditions. The comparison is interesting since, although both diseases cause gait abnormalities and balance problems, AD subjects were reported to have an increased body sway in quiet stance, which was instead decreased in PD subjects.

In the first study [51] patients underwent the Sensory Organization Test protocol, aiming to disclose whether the distraction from incongruent stimuli—and therefore the ability to shift attention—might be an important factor in determining postural instability and frequent falling. Attending to relevant sensory information while concurrently suppressing incongruent or distracting information compels substantial cortical involvement, as witnessed by the ability of patients with primary subcortical damage to perform in a similar way to healthy individuals when specifically challenged to this requirement [53]. Parkinsonian patients showed similar total fall incidence as AD patients, but the distribution was generalized across a higher number of sensory conditions, suggesting that their disability in the Sensory Organization Test could be due to difficulty in quickly changing set to match changes in balance conditions [51].

This suggestion was addressed in a companion study investigating adaptation of leg muscle activity in AD, PD patients, and age-matched healthy controls, when their postural set was influenced via changes in support conditions of holding or sitting [52]. Postural set indicates how well subjects use relevant environmental and sensory cues to prepare for or respond to an impending external threat to their balance. This ability implies changing the transmission of neural pathways on the basis of expectation, prior experience or context, to confer flexible adaptation of leg muscle activity to changes in the environment. The ability to quickly change the postural set was inferred by comparing leg muscle activity under two conditions of support (free stance versus grasping a frame, or sitting) during backward surface translations, during toes up surface rotations, and during voluntary rise to toes. Whereas AD subjects performed similarly to controls, that is, they changed postural set immediately by suppressing leg muscle activity to low levels when supported, PD subjects did not. They failed to suppress both the tibialis anterior muscle activity in voluntary rise to toes when holding and the soleus muscle activity in perturbed sitting in the first trial, and required repeated and consecutive trials in both protocols to adapt postural responses to the novel perturbation. Therefore, PD subjects had difficulty in quickly changing set in all types of tasks, suggesting a general, rather than task-specific, deficit in changing postural set. The presence of this disability in PD but not in AD subjects suggests that the influence of postural set on motor coordination operates mainly at the subcortical level, likely involving the basal ganglia [52].

Findings from the previous study also suggest that inflexible postural behavior in PD patients is not an all-or-none phenomenon, because they can still change sensorimotor set, even if slower than normal. PD difficulty in changing postural set quickly is consistent with other known motor abnormalities that cause patients to have trouble with rapid sequencing of voluntary complex movements [54]. Since the physiology of voluntary and automatic movements differs by involving distinct pattern and timing of activation of primary and nonprimary motor areas, set-dependent disability in PD patients performing voluntary movements cannot be attributed primarily to a disorder of the basal ganglia or their connections with nonprimary motor and other frontal areas. To see whether basal ganglia are the critical structures for changing set quickly, PD patients were subjected to an automatic set-changing task in which standing subjects attempted to maintain their balance during abrupt movements of the support surface [55]. Experimentally, the ability to change set was inferred by measuring the change in amplitude of automatic muscle responses in standing subjects in two different conditions: when either the direction of a surface perturbation (i.e., from backward to toe-up, sensorimotor set) or the instructions (give versus resist, cognitive set) delivered to the subject were changed. Suppression of gastrocnemius responses when toe-up rotation followed repeated backward translations indicated a change in sensorimotor set that took place immediately in the first rotation in controls while it needed several rotations to happen in PD subjects. When task instructions were changed, PD subjects had more difficulty than controls in using cognitive set to modify their postural responses, and, more specifically, they had greater difficulty in increasing their response to the “resist” than in decreasing their response to the “give” instruction. Again, these findings showed that PD difficulty in changing set is widespread and reflects a general disability rather than a task-specific problem. The authors concluded that basal ganglia are involved in a general, rather than specific, function associated with a set mechanism; that is, they do not define or coordinate action, but they rather prime or set the nervous system to achieve its goal [55].

## 5. Abnormal Background Muscle Tone 

Rigidity is a form of muscle hypertonia. It is characterized by increased stiffness experienced during passive mobilization of a limb segment, irrespective of the direction of the mobilization. It has been long been known as a cardinal sign of PD that affects both axial and limb segments [56]. Early indications of rigidity are impaired arm swing during gait and the tendency of the head to remain in line with the rest of the body during turns.

In Parkinsonian patients, rigidity contributes to altered motor control and disability. Specifically, axial rigidity reduces body rotation during sleep [57], induces abnormal head-trunk intersegmental coordination during walking and turning [58], and by altering the control of pelvis on hip rotation it affects gait speed and turning [59]. Axial rigidity is not uniform, as direct measures of torsional resistance of the longitudinal axis to twisting show that OFF-medication Parkinsonian subjects have higher rigidity in the hips than the trunk, greater hip-to-trunk torque ratio than controls, and positive correlation between scores of hip rigidity and total OFF-medication UPDRS score [60]. In this study, subjects were tested when they stood without changing body orientation relative to gravity, and their body parts fixed against rotation were only free to translate laterally within the boundaries of normal postural sway, leading investigators to conclude that axial hypertonia may underlie functional impairments of posture and locomotion in PD.

As far as it concerns axial rigidity, neck hypertonia has a major impact on walking and turning since the head must be free to move to scan surrounding environment and to steer locomotion [61]. Using a device called “twister,” which measures the resistance to passively applied torsional rotation at the neck, trunk and/or hips without constraining anterior-posterior, lateral, or vertical body position, axial tone can be quantified at the neck, the trunk, and the hip [62]. Healthy adults' axial tone is not static but involves flexible, active shortening and lengthening reactions. When the twister is applied to Parkinsonian subjects, axial rigidity is found in all segments, but it predominates at the neck [63]. Neck hypertonia, more than trunk or hip rigidity, proved to be closely related to the subjects' functional performance, suggesting that muscle tone in this area plays a critical role in the control of balance, mobility, and coordination. Indeed, vibration of neck muscles during standing causes larger postural disturbances than vibration of other muscles of the trunk and legs [64].

Neck hypertonia is usually related to the abnormal static alignment derived from stooped posture. Interestingly, in the study of Franzén and colleagues [63], flexed postural alignment measured by the item 28 of the UPDRS did not correlate with axial tone in the studied subjects nor did the increased resistance to axial rotation around the vertical axis result from limitations in the passive or active range-of-motion. Therefore, neck rigidity may be associated with stooped posture in PD, but these two abnormalities have no reciprocal causative effect.

Independence between neck rigidity and stooped posture does not necessarily mean that stooped stance cannot influence postural stability. This assumption has been challenged by assessing automatic postural responses in healthy subjects instructed either to stand upright or to assume a typical Parkinsonian posture [65]. During both conditions, subjects received “toe-up” rotational perturbations from a supporting force plate, and responses from stretched and shortened muscles were recorded, together with changes in the center of foot pressure and the center of gravity. Measures obtained in the two conditions were qualitatively compared with the typical pattern of response observed in Parkinsonian subjects, that is, large MLR and small LLR with insufficient voluntary postural corrections, and slow rate of backward displacement of the center of foot pressure and increased posterior displacement of the center of gravity. When the healthy subjects mimicked the stooped posture, they unloaded medial gastrocnemius and loaded tibialis anterior muscle. In this condition, stretch responses in the gastrocnemius were delayed, whereas LLRs in the tibialis anterior were markedly reduced. The amplitudes of both responses were reduced, and both the center of gravity and, to a lesser extent, the center of foot pressure were shifted forward. The posterior displacement of the centre of gravity and the rate of backward displacement of the centre of foot pressure were diminished, but voluntary postural corrections were normal. These observations show that healthy subjects mimicking a stooped posture reproduce some (i.e., reduced LLRs) Parkinsonian postural abnormalities but not others (i.e., increased MLRs and insufficient voluntary responses), indicating that only the latter may contribute to the balance control impairment in PD [65].

Finally, it is interesting to note that latency and amplitude of the medium- and long-latency reflexes recorded from forearm muscles in response to step change in load have been shown to be related to activated rigidity [66, 67], which also contribute to inefficient posture control. However, the fact that levodopa has no effect on axial hypertonia while it alleviates limb rigidity suggests that separate neural circuits control the tone of axial and appendicular muscle groups [60, 63].

## 6. Conclusions

Poor balance control and postural instability are among the most disabling features of PD. The sensorimotor control of posture involves a complex integration of multisensory inputs that provide a properly scaled neuromuscular response, which—taking into account the background muscle tone—results in a final motor adjustment process.

Sensory control is operated by integrating data coming from proprioceptive, vestibular, and visual channels. All or some of these systems may be dysfunctional in Parkinsonian patients. They exhibit increased dependence on visual information and are unable to maintain balance control when visual cues are absent or unreliable, or when they conflict with input coming from vestibular and proprioceptive systems. The impairment of one sensory channel may be further compounded by dysfunction in another, as it happens, for example, in the visual and vestibular systems. The latter is responsible for the fine-tuning of balance control, and if abnormal, reduces the effectiveness of visual and proprioceptive systems to provide feedback for successful balance control.

Following perturbations Parkinsonian patients usually manifest dysfunctional proprioceptive mechanisms with slowed corrective movements about the ankle joint and increased body sway. They manifest a type of postural inflexibility by activating the ankle and hip strategies simultaneously, regardless of their normal postural latencies. PD patients lack the modification of postural muscle synergies normally associated with changes in support conditions and consequently displace forward their centre of foot pressure, indicating high stiffness in ankle muscles. The increased muscle stiffness and inflexibility of postural reflexes contribute to balance control impairment.

Deficits in the timing and modulation of postural reactions are unlikely to be caused by abnormalities in kinaesthesia, but abnormal processing of proprioceptive feedback affects the subjects' ability to properly adjust the gain of their postural responses to varying balance perturbations (i.e., scaling). A recently proposed model of postural control [41]—which involves multivariate linear feedback model simulations–proved to be able to reproduce postural responses in young, elderly, and PD patients for a wide range of surface perturbations. The model quantifies the patients' postural disturbance as smaller than normal ankle feedback gain, larger than normal hip feedback gains, and an inflexible selection of feedback gain as the perturbation conditions change.

Stability and mobility in functional motor activities depend on the precise regulation of phasic and tonic muscular activity that is carried out automatically, without conscious awareness. In Parkinsonian patients rigidity interferes with this automatic activity, especially the axial component. Among rigid axial regions, the neck appears to play an important role in the control of balance, mobility, and coordination. Indeed, many falls associated with sudden changes in postural orientation, such as turning, are thought to be due to inflexible control of axial postural tone.

From the time of Purdon-Martin's clinical observations that Parkinsonian subjects lack the righting automatic postural responses when pushed and that they exceed normal limits of stability after body tilt, some aspects of the pathophysiology of postural instability in PD began to be understood. It is now clear that PD impairs posture and balance control by slowing and weakening automatic postural responses to perturbation, fragmenting the muscular activation that generates these responses, and leads to cocontraction, changing ankle muscle strength and stiffness, altering amplitude scaling of the motor response, inducing distorted perception of stability limits, and interfering with the generation of automatic responses owing to rigidity, especially involving the axial body regions. Other components of the complex process that exerts the sensorimotor control of posture may be dysfunctional in PD, some of them being unique to each patient in the situation of disequilibrium. Among these components there are poor control of voluntary movement, motor side effects of medication (dyskinesias), autonomic abnormalities, and superimposed age-related changes in neuromuscular and musculoskeletal systems.

## References

[1] Bohnen N. I., Müller M. L. T. M., Koeppe R. A. (2009). History of falls in Parkinson disease is associated with reduced cholinergic activity. *Neurology*.

[2] Berg D., Lang A. E., Postuma R. B. (2013). Changing the research criteria for the diagnosis of Parkinson's disease: obstacles and opportunities. *The Lancet Neurology*.

[3] Martin J. P. (1967). *The Basal Ganglia and Posture*.

[4] Doherty K. M., van de Warrenburg B. P., Peralta M. C. (2011). Postural deformities in Parkinson's disease. *The Lancet Neurology*.

[5] Beckley D. J., Bloem B. R., van Dijk J. G., Roos R. A. C., Remler M. P. (1991). Electrophysiological correlates of postural instability in Parkinson's disease. *Electroencephalography and Clinical Neurophysiology*.

[6] Horak F. B., Nutt J. G., Nashner L. M. (1992). Postural inflexibility in parkinsonian subjects. *Journal of the Neurological Sciences*.

[7] Suchowersky O., Reich S., Perlmutter J., Zesiewicz T., Gronseth G., Weiner W. J. (2006). Practice parameter: diagnosis and prognosis of new onset Parkinson disease (an evidence-based review): Report of the Quality Standards Subcommittee of the American Academy of Neurology. *Neurology*.

[8] Bronstein A. M., Brandt T., Woollacott M. (1996). *Clinical Disorders of Balance, Posture and Gait*.

[9] Allum J. H. J., Bloem B. R., Carpenter M. G., Hulliger M., Hadders-Algra M. (1998). Proprioceptive control of posture: a review of new concepts. *Gait and Posture*.

[10] Proske U. (2006). Kinesthesia: the role of muscle receptors. *Muscle and Nerve*.

[11] Proske U., Gandevia S. C. (2009). The kinaesthetic senses. *The Journal of Physiology*.

[12] Loram I. D., Lakie M., di Giulio I., Maganaris C. N. (2009). The consequences of short-range stiffness and fluctuating muscle activity for proprioception of postural joint rotations: the relevance to human standing. *Journal of Neurophysiology*.

[13] Peterka R. J. (2002). Sensorimotor integration in human postural control. *Journal of Neurophysiology*.

[14] Mergner T., Siebold C., Schweigart G., Becker W. (1991). Human perception of horizontal trunk and head rotation in space during vestibular and neck stimulation. *Experimental Brain Research*.

[15] Onofrj M., Thomas A., D'Andreamatteo G. (2002). Incidence of RBD and hallucination in patients affected by Parkinson's disease: 8-year follow-up. *Neurological Sciences*.

[16] Barbato L., Rinalduzzi S., Laurenti M., Ruggieri S., Accornero N. (1994). Color VEPs in Parkinson's disease. *Electroencephalography and Clinical Neurophysiology*.

[17] Armstrong R. A. (2011). Visual symptoms in Parkinson's disease. *Parkinson's Disease*.

[18] Rinalduzzi S., Capozza M., Accornero N., Manfredi G. W., Calandriello L., Meco G. (1995). Postural strategies in Parkinson and cerebellar patients. *Italian Journal of Neurological Sciences*.

[19] Bronstein A. M., Hood J. D., Gresty M. A., Panagi C. (1990). Visual control of balance in cerebellar and Parkinsonian syndromes. *Brain*.

[20] Vaugoyeau M., Viel S., Assaiante C., Amblard B., Azulay J. P. (2007). Impaired vertical postural control and proprioceptive integration deficits in Parkinson's disease. *Neuroscience*.

[21] Poizner H., Feldman A. G., Levin M. F. (2000). The timing of arm-trunk coordination is deficient and vision-dependent in Parkinson's patients during reaching movements. *Experimental Brain Research*.

[22] Klockgether T., Dichgans J. (1994). Visual control of arm movement in Parkinson's disease. *Movement Disorders*.

[23] Jackson S. R., Jackson G. M., Harrison J., Henderson L., Kennard C. (1995). The internal control of action and Parkinson's disease: a kinematic analysis of visually-guided and memory-guided prehension movements. *Experimental Brain Research*.

[24] Rinalduzzi S., Cipriani A. M., Capozza M., Accornero N. (2011). Postural responses to low-intensity, short-duration, galvanic vestibular stimulation as a possible differential diagnostic procedure. *Acta Neurologica Scandinavica*.

[25] Pastor M. A., Day B. L., Marsden C. D. (1993). Vestibular induced postural responses in Parkinson's disease. *Brain*.

[26] Pal S., Rosengren S. M., Colebatch J. G. (2009). Stochastic galvanic vestibular stimulation produces a small reduction in sway in Parkinson's disease. *Journal of Vestibular Research: Equilibrium and Orientation*.

[27] Maschke M., Gomez C. M., Tuite P. J., Konczak J. (2003). Dysfunction of the basal ganglia, but not the cerebellum, impairs kinaesthesia. *Brain*.

[28] Tagliabue M., Ferrigno G., Horak F. (2009). Effects of Parkinson's disease on proprioceptive control of posture and reaching while standing. *Neuroscience*.

[29] Wright W. G., Gurfinkel V., King L., Horak F. (2007). Parkinson's disease shows perceptuomotor asymmetry unrelated to motor symptoms. *Neuroscience Letters*.

[30] Konczak J., Krawczewski K., Tuite P., Maschke M. (2007). The perception of passive motion in Parkinson's disease. *Journal of Neurology*.

[31] Wright W. G., Gurfinkel V. S., King L. A., Nutt J. G., Cordo P. J., Horak F. B. (2010). Axial kinesthesia is impaired in Parkinson's disease: effects of levodopa. *Experimental Neurology*.

[32] Diener H. C., Bootz F., Dichgans J., Bruzek W. (1983). Variability of postural “reflexes” in humans. *Experimental Brain Research*.

[33] Diener H. C., Dichgans J., Bootz F., Bacher M. (1984). Early stabilization of human posture after a sudden disturbance: influence of rate and amplitude of displacement. *Experimental Brain Research*.

[34] Horak F. B., Nashner L. M. (1986). Central programming of postural movements: adaptation to altered support-surface configurations. *Journal of Neurophysiology*.

[35] Schieppati M., Nardone A. (1991). Free and supported stance in Parkinson's disease. *Brain*.

[36] Scholz E., Diener H. C., Noth J., Friedemann H., Dichgans J., Bacher M. (1987). Medium and long latency EMG responses in leg muscles: Parkinson's disease. *Journal of Neurology, Neurosurgery, and Psychiatry*.

[37] Berardelli A., Dick J. P. R., Rothwell J. C., Day B. L., Marsden C. D. (1986). Scaling of the size of the first agonist EMG burst during rapid wrist movements in patients with Parkinson's disease. *Journal of Neurology Neurosurgery and Psychiatry*.

[38] Beckley D. J., Bloem B. R., Remler M. P. (1993). Impaired scaling of long latency postural reflexes in patients with Parkinson's disease. *Electroencephalography and Clinical Neurophysiology*.

[39] Valkovič P., Krafczyk S., Šaling M., Benetin J., Bötzel K. (2006). Postural reactions to neck vibration in Parkinson's disease. *Movement Disorders*.

[40] Valkovič P., Krafczyk S., Bötzel K. (2006). Postural reactions to soleus muscle vibration in Parkinson's disease: scaling deteriorates as disease progresses. *Neuroscience Letters*.

[41] Kim S., Horak F. B., Carlson-Kuhta P., Park S. (2009). Postural feedback scaling deficits in Parkinson's disease. *Journal of Neurophysiology*.

[42] Dietz V., Berger W., Horstmann G. A. (1988). Posture in Parkinson's disease: impairment of reflexes and programming. *Annals of Neurology*.

[43] Bloem B. R., Beckley D. J., Gert van Dijk J., Zwinderman A. H., Remler M. P., Roos R. A. C. (1996). Influence of dopaminergic medication on automatic postural responses and balance impairment in Parkinson's disease. *Movement Disorders*.

[44] Horak F. B., Frank J., Nutt J. (1996). Effects of dopamine on postural control in parkinsonian subjects: scaling, set, and tone. *Journal of Neurophysiology*.

[45] Schmidt R. A. (1982). *Motor Control and Learning: A Behavioral Emphasis*.

[46] Greene P. H. (1972). Problems of organization of motor systems. *Progress in Theoretical Biology*.

[47] Brooks V. B. (1984). Cerebellar function in motor control. *Human Neurobiology*.

[48] Evarts E. V. (1975). The third Stevenson lecture. Changing concepts of central control of movement. *Canadian Journal of Physiology and Pharmacology*.

[49] Diener H. C., Horak F. B., Nashner L. M. (1988). Influence of stimulus parameters on human postural responses. *Journal of Neurophysiology*.

[50] Horak F. B., Diener H. C., Nashner L. M. (1989). Influence of central set on human postural responses. *Journal of Neurophysiology*.

[51] Chong R. K. Y., Horak F. B., Frank J., Kaye J. (1999). Sensory organization for balance: specific deficits in Alzheimer's but not in Parkinson's disease. *Journals of Gerontology—Series A Biological Sciences and Medical Sciences*.

[52] Chong R. K. Y., Jones C. L., Horak F. B. (1999). Postural set for balance control is normal in Alzheimer's but not in Parkinson's disease. *Journals of Gerontology A: Biological Sciences and Medical Sciences*.

[53] Spieler D. H., Balota D. A., Faust M. E. (1996). Stroop performance in healthy younger and older adults and in individuals with dementia of the AIzheimer's type. *Journal of Experimental Psychology: Human Perception and Performance*.

[54] Currà A., Berardelli A., Agostino R. (1997). Performance of sequential arm movements with and without advance knowledge of motor pathways in Parkinson's disease. *Movement Disorders*.

[55] Chong R. K., Horak F. B., Woollacott M. H. (2000). Parkinson's disease impairs the ability to change set quickly. *Journal of the Neurological Sciences*.

[56] Foster M. A. (1892). The central nervous system. *Text Book of Physiology*.

[57] Nutt J. G., Woodward W. R., Carter J. H., Gancher S. T. (1992). Effect of long-term therapy on the pharmacodynamics of levodopa: relation to on-off phenomenon. *Archives of Neurology*.

[58] Mesure S., Azulay J. P., Pouget J., Amblard B. (1999). Strategies of segmental stabilization during gait in Parkinson's disease. *Experimental Brain Research*.

[59] Vaugoyeau M., Viallet F., Aurenty R., Assaiante C., Mesure S., Massion J. (2006). Axial rotation in Parkinson's disease. *Journal of Neurology, Neurosurgery and Psychiatry*.

[60] Wright W. G., Gurfinkel V. S., Nutt J., Horak F. B., Cordo P. J. (2007). Axial hypertonicity in Parkinson's disease: direct measurements of trunk and hip torque. *Experimental Neurology*.

[61] Paquette C., Paquet N., Fung J. (2006). Aging affects coordination of rapid head motions with trunk and pelvis movements during standing and walking. *Gait & Posture*.

[62] Gurfinkel V., Cacciatore T. W., Cordo P., Horak F., Nutt J., Skoss R. (2006). Postural muscle tone in the body axis of healthy humans. *Journal of Neurophysiology*.

[63] Franzén E., Paquette C., Gurfinkel V. S., Cordo P. J., Nutt J. G., Horak F. B. (2009). Reduced performance in balance, walking and turning tasks is associated with increased neck tone in Parkinson's disease. *Experimental Neurology*.

[64] Gregoric M., Lavric A. (1977). Statokinesimetric analysis of the postural control in Parkinsonism. *Agressologie*.

[65] Bloem B. R., Beckley D. J., Van Dijk J. G. (1999). Are automatic postural responses in patients with Parkinson's disease abnormal due to their stooped posture?. *Experimental Brain Research*.

[66] Mortimer J. A., Webster D. D. (1979). Evidence for a quantitative association between EMG stretch responses and Parkinsonian rigidity. *Brain Research*.

[67] Tatton W. G., Lee R. G. (1975). Evidence for abnormal long loop reflexes in rigid Parkinsonian patients. *Brain Research*.

